# Comparison of outcomes of percutaneous coronary intervention on proximal versus non-proximal left anterior descending coronary artery, proximal left circumflex, and proximal right coronary artery: A cross-sectional study

**DOI:** 10.1186/1471-2261-7-7

**Published:** 2007-03-04

**Authors:** Mohammad Alidoosti, Mojtaba Salarifar, Ali Mohammad Haji Zeinali, Seyed Ebrahim Kassaian, Maria Raissi Dehkordi

**Affiliations:** 1 Department of Interventional Cardiology, Tehran Heart Center, Medical Sciences/University of Tehran. Jalal Al Ahmad and North Karegar Cross PO Box: 1411713138 Tehran, Iran; 2 Research Department, Tehran Heart Center, Medical Sciences/University of Tehran, Jalal Al Ahmad and North Karegar Cross. PO Box: 1411713138 Tehran, Iran

## Abstract

**Background:**

Previous studies have shown that lesions in proximal left anterior descending coronary artery (LAD) may develop more restenosis after balloon angioplasty than lesions in other coronary segments. However, stenting seems to have reduced this gap. In this study, we compared outcomes of percutaneous coronary intervention (PCI) on proximal LAD versus proximal left circumflex (LCX) or right coronary artery (RCA) and proximal versus non-proximal LAD.

**Methods:**

From 1737 patients undergoing PCI between March 2004 and 2005, those with cardiogenic shock, primary PCI, total occlusions, and multivessel or multi-lesion PCI were excluded. Baseline characteristics and in-hospital outcomes were compared in 408 patients with PCI on proximal LAD versus 133 patients with PCI on proximal LCX/RCA (study I) and 244 patients with PCI on non-proximal LAD (study II). From our study populations, 449 patients in study I and 549 patients in study II participated in complete follow-up programs, and long-term PCI outcomes were compared within these groups. The statistical methods included Chi-square or Fisher's exact test, student's t-test, stratification methods, multivariate logistic regression and Cox proportional hazards model.

**Results:**

In the proximal LAD vs. proximal LCX/RCA groups, smoking and multivessel disease were less frequent and drug-eluting stents were used more often (p = 0.01, p < 0.001, and p < 0.001, respectively). Patients had longer and smaller-diameter stents (p = 0.009, p < 0.001, respectively). In the proximal vs. non-proximal LAD groups, multivessel disease was less frequent (p = 0.05). Patients had larger reference vessel diameters (p < 0.001) and were more frequently treated with stents, especially direct stenting technique (p < 0.001). Angiographic success rate was higher in the proximal LAD versus proximal LCX/RCA and non-proximal LAD groups (p = 0.004 and p = 0.05, respectively). In long-term follow-up, major adverse cardiac events showed no difference. After statistical adjustment for significant demographic, angiographic or procedural characteristics, long-term PCI outcomes were still similar in the proximal LAD versus proximal LCX/RCA and non-proximal LAD groups.

**Conclusion:**

Despite the known worse prognosis of proximal LAD lesions, in the era of stenting, our long-term outcomes were similar in patients with PCI on proximal LAD versus proximal LCX/RCA and non-proximal LAD. Furthermore, we had better angiographic success rates in patients with PCI on proximal LAD.

## Background

Stenosis of the proximal segment of the left anterior descending coronary artery (LAD) is a special subgroup of coronary artery disease (CAD), given the high-risk profile that these lesions have alone or in the context of multivessel disease [1,2]. Patients with proximal LAD occlusion in association with low left ventricular ejection fraction have been reported to have 19-fold higher mortality than the general population [3].

The data from previous studies suggest that the outcome after revascularization depends on the distribution of prerevascularization coronary artery disease. In that regard, the severity and location of LAD involvement have been identified as important determinants of outcome in patients with CAD [4]. This lesion carries a high risk of restenosis after balloon angioplasty with reported patency rate at six month-follow-up of approximately 75% [5]. However, some studies have shown that stenting may reduce the rate of restenosis in these lesions in comparison with balloon angioplasty [6]. Specifically, drug-eluting stents have been shown to be related to significantly reduced restenosis rates compared to bare metal stents [7,8].

Numerous studies have investigated the outcome of percutaneous coronary intervention (PCI) and bypass surgery in patients with LAD stenosis [9-11]. Generally, the presence of a proximal LAD stenosis has swayed the cardiologists to refer the patients to coronary artery bypass grafting (CABG) presuming these lesions have an overall higher rate of restenosis than proximal left circumflex or right coronary artery [12]. However, according to the American College of Cardiology/American Heart Association, only type B2/C lesions of the LAD carry a high risk of target vessel revascularization (TVR) and morbidity [13] and surgery has been proposed as a better option only for such lesions [14]. According to the 2003 report from the Iranian Statistical Center, 14100 PCI procedures were performed in 50 hospitals in Iran. Of these, approximately 1700 procedures were carried out in Tehran Heart Center (THC). Because of the clinical significance of the lesions located in proximal LAD, our objective in this study was to evaluate the procedural results, in-hospital and long-term clinical outcomes of PCI on proximal LAD, proximal left circumflex (LCX) or right coronary artery(RCA), and non-proximal LAD.

## Methods

### Study population

From a total of 1737 consecutive patients undergoing PCI at THC between March 2004 and March 2005, a patient cohort with PCI for de novo narrowing in the proximal segments of coronary arteries or non-proximal LAD were included in the study. Patients with cardiogenic shock, primary PCI, total occlusions, and multivessel or multi-lesion PCI were excluded. Among the 785 remaining patients: 408 had PCI on proximal LAD, 133 underwent PCI on proximal LCX or RCA, and 244 patients were treated with PCI on non-proximal LAD. In study I (n = 541), we compared the outcomes of PCI on proximal LAD versus proximal LCX/RCA. In study II (n = 652), the outcome of PCI on proximal LAD versus non-proximal LAD were compared. Baseline, clinical, angiographic, and procedural characteristics plus in-hospital outcomes were obtained by research physicians and entered into a computerized database by computer operators. Finally, 449 patients in study I and 549 patients in study II agreed to participate in follow-up programs. They were monitored by cardiologists 1, 5, and 9 months post angioplasty and once a year thereafter. Patient follow up was done in the clinic or by formal telephone interviews. Clinical data obtained were: major adverse cardiac events (MACE), including cardiac death, non-fatal myocardial infarction (MI), and TVR (CABG or repeated PCI). The data were recorded in data sheets and later transferred into a computerized database. This study was approved by the THC Ethics Committee. Informed consent was obtained from patients before enrolment into this study.

### Definitions

Patients already taking hypertensive medication or those whose average of two blood pressure readings at least five minutes apart in the sitting posture was ≥ 140/90 mmHg were labeled as hypertensive [15]. Patients with a history of taking antihyperlipidemic drugs, total cholesterol ≥ 200 mg/dl or low density lipoprotein ≥ 130 mg/dl were defined as hypercholestrolemic [16]. Diabetes mellitus was diagnosed to be present if a patient had a definite history of treated diabetes, fasting plasma glucose ≥ 126 mg/dl or two-hour post-load glucose ≥ 200 mg/dl, based on the guidelines of the American Diabetes Association [17]. Angina symptoms were defined according to the classification of the Canadian Cardiovascular Society [18]. Lesion types were noted according to the American College of Cardiology/American Heart Association (ACC/AHA) lesion characteristics classification [19]. Left ventricular ejection fraction was obtained from cardiac catheterization ventriculograms. Q wave MI was defined as the presence of new Q waves in post-procedure electrocardiograms with a 3-fold increase in MB fraction of creatinine kinase. Non-Q wave MI was defined as a 3-fold increase in MB fraction of creatinine kinase without the development of new Q waves [20]. Angiographic success was defined as residual stenosis <20% for stenting and <50% for balloon angioplasty plus normal Thrombolysis in Myocardial Infarction (TIMI) 3 flow. Procedural success was defined as angiographic success without major complications (death, MI, emergency CABG or PCI) during hospitalization [19]. Major adverse cardiac events were defined as the presence of cardiac death, MI, and TVR during follow-up. TVR was defined as ischemia-driven repeat percutaneous intervention or bypass surgery of the target vessel. Target lesion revascularization (TLR) was defined as ischemia-driven repeat percutaneous intervention of the target lesion or bypass surgery of the target vessel [21]. Proximal stenosis was defined as stenosis located between the origin of the vessel to the takeoff of the first side branch (first diagonal branch for LAD).

### Coronary procedures

Patients received 325 mg of Aspirin before the procedure, which was continued indefinitely. Coronary stenting was performed by standard methods. After stent placement, ticlopidine (250 mg twice daily) or clopidogrel (75 mg once daily) was given routinely for 4 weeks for bare metal stents and 6 to 12 months for drug-eluting stents. Glycoprotein IIb/IIIa blocker was not used in any patient. Angiographic findings such as vessel dimensions, pre-and post-procedural stenoses, lesion length, and TIMI flow grade were determined by visual estimation using the guiding catheter as a reference object for calibration. The angiographic characteristics were also further analyzed by an independent interventional cardiologist not involved in the procedure and checked for inter-observer agreement (kappa coefficient = 0.91, 95% CI = 0.85–0.97).

### Statistical analysis

Statistical testing was performed by chi-square test or Fisher's exact test (2-tailed) for categorical variables. Student's t-test was used for comparison of continuous variables. Because of the imbalances in some baseline characteristics, the main study results were confirmed by means of stratified analyses and multivariate logistic regression models. Stratification was performed for variables that either had significant imbalance between the groups (p < 0.05), or were clinically important. These variables included sex, diabetes mellitus, prior PCI, use of drug-eluting stents, angulated segments, stent length, and stent diameter. Furthermore, we constructed multivariate logistic regression models with selected variables that had p values < 0.05 in univariate analysis (sex and use of drug-eluting stents were also included). The p values of these variables are marked in bold l in tables 1 and 2. Long-term MACE modeling was performed with Cox proportional hazards model. The statistical analyses were performed with SAS software version 9.1. Differences were considered statistically significant at p values < 0.05.

## Results

### Baseline characteristics

Table 1 represents selected baseline characteristics of patients treated for proximal stenoses of major epicardial arteries and non-proximal LAD. Current smoking and prior non-target vessel PCI had a lower frequency in patients with PCI on proximal LAD versus LCX or RCA. However, in patients with PCI on proximal versus non-proximal LAD, the baseline demographic characteristics did not differ. Mean Ejection Fraction was 53.54 ± 9.45% in the proximal LAD versus 53.12 ± 10.24% and 54.35 ± 9.22% in the proximal LCX/RCA and non-proximal LAD groups (p = NS), respectively. In the proximal LAD versus proximal LCX/RCA and non-proximal LAD, the frequency of unstable angina (33.7% vs. 34.8% and 33.7%) and prior MI (33.8% vs. 28.6% and 29.5%) had no statistically significant difference (P = NS).

### Angiographic and procedural characteristics (Table 2)

Frequency of type B2/C lesions was similar in the proximal LAD vs. proximal LCX/RCA and non-proximal LAD groups: (42.8% vs.43.8% and 47.8, p = NS). Multivessel disease and angulated segments were less frequent in the proximal LAD group. In the proximal LAD versus proximal LCX/RCA group, lesions were longer (16.89 ± 8.1 mm versus 14.68 ± 7.38 mm, p = 0.007) and had lower frequency of proximal segment tortuosity (p = 0.03). However, percentage of lesions smaller than 3 mm in diameter did not differ. On the other hand, in the proximal versus non-proximal LAD group, expectedly, lesions had larger reference vessel diameters. Stents were used in 99.8% of patients in the proximal LAD versus 98.5% in the proximal LCX/RCA (p = NS) and 96.7% in the non-proximal LAD groups (p = 0.002). Pre- and post-procedural stenosis percentages did not differ significantly in the proximal LAD versus proximal LCX/RCA and non-proximal LAD groups (89.44 ± 8.65% versus 89.15 ± 9.37% and 89.96 ± 8.28%; 0.65 ± 3.24% versus 2.75 ± 13.69% and 1.11 ± 4.51%, p = NS). Patients with PCI on proximal LAD versus proximal LCX/RCA were more commonly treated with drug-eluting stents and had longer stents with smaller diameters. Patients with proximal versus non-proximal LAD stenosis were more often treated with stents, especially the direct stenting technique.

### In-hospital and late clinical outcomes

**Figure 1:**
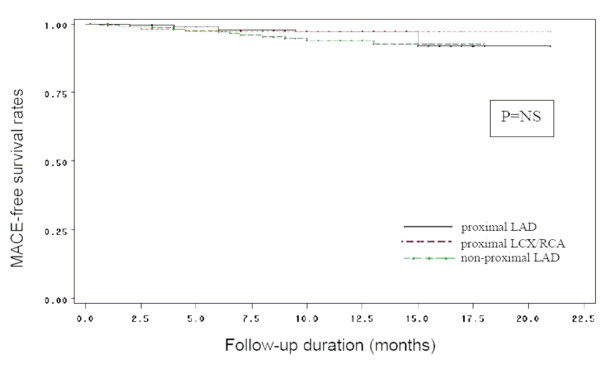
**MACE-free survival rates in the proximal LAD versus proximal LCX/RCA and non-proximal LAD groups**. MACE = Major adverse cardiac events.

In the proximal LAD group, procedures were angiographically successful in 100% of cases, which was significantly greater than the other two groups. In-hospital events, which occurred only as non-Q wave MI in our cohort, were observed in 8 (2%) in the proximal LAD group, while only 1 patient in the proximal LCX/RCA and non-proximal LAD group (0.8% and 0.4% respectively) displayed such symptoms(P = NS). Procedural success rates were similar in both comparisons (table 3). Mean follow-up duration was 10.81 ± 3.69 months in total population. Major adverse cardiac events at 9-month follow-up, including cardiac death, MI, and TVR (CABG or repeated PCI) were not significantly different in the proximal LAD versus the other two groups (table 3). After stratification for factors that either had significant imbalance between the groups or were clinically important, the MACE rates in the proximal LAD versus proximal LCX/RCA and non-proximal LAD groups were still comparable (table 4). Similarly, in multivariate logistic regression models, there was no difference in MACE rates in the proximal LAD versus proximal LCX/RCA (p = 0.85, 95% CI = 0.19–3.89, OR = 0.87) and proximal versus non-proximal LAD groups (p = 0.66, 95% CI = 0.29–2.1, OR = 0.8). Cox proportional hazards model also did not show any difference in MACE-free survival rates at long-term follow-up (figure 1).

## Discussion

The main finding that emerges from this study is that long-term outcomes of PCI including MACE in patients with proximal LAD stenosis are similar to patients with proximal LCX or RCA and non-proximal LAD stenosis. Also worth noting from our findings was that angiographic success was higher in patients with proximal LAD stenosis.

Management of proximal coronary artery disease is important due to the large areas of myocardium that lie downstream of the stenoses. The proximal LAD artery stenosis represents the most important proximal site for obstructive coronary artery after left main stem lesion disease, as it supplies 40%–50% of the total left ventricular myocardium and could result in ischemia to a large area of myocardium [22,23]. Moreover, Patients with a critical stenosis of the proximal LAD segment are particularly prone to adverse effects of MI and, therefore, require a safe and long-term effective method of treatment [24].

Previous studies have demonstrated that interventional treatment produces are more beneficial than pharmacological treatment in severe proximal LAD artery disease [6]. However, The results of nonstenting PCI to proximal LAD stenoses has been less satisfactory than procedures in the proximal right/circumflex coronary arteries due to higher rates of restenosis in this area [12,25,26]. Stenting leads to better clinical outcomes than percutaneous transluminal coronary angioplasty in the treatment of isolated lesions of proximal LAD by reducing the risk of restenosis by about 50% [3]. In fact, stenting of lesions in the proximal LAD is as effective and safe as treatment of lesions located in distal LAD. Therefore, in the current stenting era, location of the lesion in the LAD is not predictive of worse outcome. Furthermore, when stenting is feasible, it may not be taken into account in the choice of revascularization strategy [27]. However, the re-intervention rates have been higher after bare-metal stenting than bypass surgery of the proximal LAD [28]. With the advent of drug-eluting stents, a reduction has been achieved in restenosis rates after stenting in proximal LAD. This in turn has narrowed the "reintervention gap" between drug-eluting stents enough to eliminate the major advantage of bypass surgery for the treatment of LAD disease [7].

In our study, no significant difference was observed in rates of major adverse cardiac events during long-term follow-up in the proximal LAD versus the other two groups. Even after adjusting for factors such as stents, particularly drug-eluting stents, which were used most frequently in the proximal LAD group, no statistical difference was detectible. MACE-free survival rates were also similar between the groups (figure 1). Before our study, Ashby et al had studied the outcomes of stenting in proximal LAD versus proximal LCX/RCA [12]. In their study, multivariate analysis was conducted to adjust these groups for some baseline differences. However, the main endpoint of this study i.e. rate of TLR, was still similar in both groups in patients treated with stents. Before that, The Stent Restenosis (STRESS) study had shown that irrespective of the procedure used (balloon angioplasty or stenting), the most important predictors of the larger luminal diameter at follow-up were the size of the luminal diameter after the procedure, the initial reference vessel diameter, and the location of a lesion in a vessel other than LAD [29].

As a secondary aim, we found that angiographic success rate was higher in patients with proximal LAD stenoses. This may be attributed to better accessibility of lesions and higher frequency of stent use in the proximal LAD group. However, because of the paucity of procedural failure rates, this effect could not be proven by means of statistical analyses.

One of the limitations of this study was the relatively small sample size of patients who had developed complications, which made it difficult to determine the predictors of MACE from the multivariate analysis. On the other hand, we had to exclude patients with multi-lesion or multivessel percutaneous coronary intervention, because otherwise we would not have been able to attribute MACE to a particular lesion site.

## Conclusion

In our study, there was no difference in the short- and long-term clinical outcomes of PCI (MACE and its components) on proximal LAD versus proximal parts of other major epicardial arteries and non-proximal LAD. However, acute angiographic success rate was higher in patients with proximal LAD lesions. After adjusting the groups for significant baseline differences, there was still no difference in clinical outcomes between these groups.

## Competing interests

The author(s) declare that they have no competing interests.

## Authors' contributions

MA designed the study, contributed substantially to the interpretation of data and progression of work, and gave the final approval for publication. MRD conducted the data collection and statistical analyses, and participated in writing of the paper. MA, SEK, AHZ, and MS equally performed the angioplasty procedures and contributed to the interpretation of results. All authors read and approved the final manuscript.

## Pre-publication history

The pre-publication history for this paper can be accessed here:



## Figures and Tables

**Table 1:** Selected baseline characteristics in the Proximal LAD versus Proximal LCX/RCA and Non-proximal LAD groups

	**Proximal LAD n = 408**	**Proximal LCX/RCA n = 133**	P value (study I) *	**Non-proximal LAD n = 244**	P value (study II) **
Age(yrs) (mean ± SD)	55.36 ± 10.87	56.94 ± 9.7	0.14	55.6 ± 10.88	0.78
Male	285(69.9%)	104(78.2%)	**0.06**	163(66.8%)	0.41
Diabetes Mellitus	76(19.1%)	33(25.4%)	0. 12	58 (24%)	0.14
Family history of CAD	89(21.8%)	21(15.8%)	0.13	54(22.1%)	0.91
hypercholesterolemia	171(43%)	59(45.4%)	0.63	107(44.2%)	0.76
Current smoker	157(38.5%)	68(51.1%)	**0.01**	86(35.2%)	0.41
hypertension	132(33.2%)	38(29.2%)	0.4	92(38%)	0.21
Myocardial infarction	138 (33.8%)	38 (28.6%)	0.26	72 (29.5%)	0.25
Prior PCI (Non-target vessel)	15(3.7%)	12(9%)	**0.01**	7(2.9%)	0.58
Prior CABG	2(0.5%)	2(1.5%)	0.24	1(0.4%)	>0.999

CAD = coronary artery disease

PCI = percutaneous coronary intervention

CABG = coronary artery bypass grafting

* Proximal LAD group vs proximal LCX/RCA group

** Proximal LAD group vs non-proximal LAD group

Variables with p values that are marked in bold were entered into multivariate analysis.

**Table 2:** Angiographic and procedural characteristics in the Proximal LAD versus Proximal LCX/RCA and Non-proximal LAD groups

	**Proximal LAD n = 408**	**Proximal LCX/RCA n = 133**	P value (study I) *	**Non-proximal LAD n = 244**	P value (study II) **
**Angiographic characteristics**					
Multivessel disease	110 (33.1%)	64 (61.5%)	**<0.001**	85 (41.5%)	**0.05**
Type B2/C	169 (42.8%)	56 (43.8%)	0.85	110 (47.8%)	0.22
Angulated segments (>45°)	9 (2.2%)	18 (13.5%)	**<0.001**	17 (7%)	**0.003**
Tubular lesions	221(54.2%)	53 (39.8%)	0.004	118 (48.4%)	0.15
Proximal tortuoisity	2 (0.5%)	4 (3%)	0.035	2 (0.8%)	0.6
RVD (<3 mm)	85 (21.6%)	21 (16.2%)	0.18	109 (46.6%)	**<0.001**
**Procedural characteristics**					
Direct stenting technique	251(61.5%)	82 (62.6%)	0.33	122(50.4%)	**<0.001**
Drug-eluting stents	97 (24.3%)	9 (7.2%)	**<0.001**	39 (17.2%)	**0.079**
Stent length(mm), mean ± SD	18.48 ± 6.79	16.68 ± 6.69	**0.009**	18.54 ± 6.77	0.9
Stent diameter(mm), mean ± SD	3.08 ± 6.79	3.23 ± 0.39	**<0.001**	2.88 ± 6.76	**<0.001**

RVD = reference vessel diameter (estimated visually)

* Proximal LAD group vs Proximal LCX/RCA group

** Proximal LAD group vs Non-proximal LAD group

Variables with p values that are marked in bold were entered into multivariate analysis.

**Table 3:** In-hospital and late outcomes of PCI in the Proximal LAD versus Proximal LCX/RCA and Non-proximal LAD groups

	**Proximal LAD n (%)**	**Proximal LCX/RCA n (%)**	P value (study I) *	**Non-proximal LAD n (%)**	P value (study II) **
**In-hospital outcomes**	**n = 408**	**n = 133**		**n = 244**	
Angiographic Success	408 (100%)	129 (97%)	0.004	241 (98.8%)	0.05
Procedural Success	400 (98%)	128 (96.2%)	0.10	240 (98.4%)	>0.999
In-hospital Events	8 (2%)	1 (0.8%)	0.45	1 (0.4%)	0.27
**9-month follow-up**	**n = 338**	**n = 111**		**n = 211**	
Major adverse cardiac events	11 (3.3%)	3 (2.7%)	>0.999	12 (5.7%)	0.17
Cardiac death	1 (0.3%)	1 (0.9%)	0.43	2 (0.9%)	0.56
Non-fatal MI	4(1.2%)	1 (0.9%)	>0.999	2 (0.9%)	>0.999
TVR	6 (1.8%)	2 (1.8%)	>0.999	7 (3.3%)	0.17
TLR	2 (0.6%)	1 (0.9%)	0.57	2 (0.9%)	0.63
CABG	4 (1.2%)	1 (0.9%)	>0.999	5 (2.4%)	0.29

MI = myocardial infarction

CABG = coronary artery bypass grafting

TVR = target vessel revascularization

TLR = target lesion revascularization

* Proximal LAD group vs Proximal LCX/RCA group

** Proximal LAD group vs Non-proximal LAD group

**Table 4:** Rate of long-term follow-up Major adverse cardiac events and Odds ratios in the patient subgroups

	**Proximal LAD **No./total (%)	**Proximal LCX/RCA **No./total (%)	Odds Ratio* (95% CI) Study I	**Non-proximal LAD **No./total(%)	Odds Ratio** (95% CI) Study II
**Sex**					
male	7/235 (3.2)	3/88(3.4)	0.9(0.2–3.4)	9/143(6.3)	0.5 (0.2–1.2)
female	4/103 (3.9)	0/23	-	3/68(4.4)	0.9(0.2–4.0)
**Diabetes mellitus**					
Yes	1/66(1.5)	0/28	-	4/52(7.7)	0.2(0.1–1.7)
No	10/373(3.6)	3/82(3.7)	1.0(0.3–3.8)	8/157(5.1)	0.7(0.3–1.9)
**Prior PCI**					
Yes	0/12	0/11	-	0/6	-
No	11/326(3.3)	3/100(3.0)	1.1(0.4–4.1)	12/205(5.9)	0.6(0.2–1.3)
**Drug-eluting stents**					
Yes	2/83(2.4)	0/6	-	1/37(2.7)	0.8(0.1–9.5)
No	9/260(3.5)	3/100(3.0)	1.2(0.4–4.5)	11/169(6.5)	0.6(0.2–1.4)
**Angulated segments**					
Yes	0/7	0/16	-	0/15	-
No	11/331 (3.3)	3/95(3.2)	1.1(0.3–3.8)	12/196(6.2)	0.5(0.3–1.2)
**Stent length**					
<20 mm	10/270(3.7)	3/94(3.2)	1.2(0.4–4.3)	8/146(5.4)	0.7(0.3–1.8)
>20 mm	1/68(1.5)	0/1	-	4/55(7.3)	0.2(0.1–1.7)
**Stent diameter**					
<3 mm	6/73(8.2)	0/15	-	7/97(7.2)	1.4(0.4–4.4)
>3 mm	5/260(1.9)	3/98(3.1)	0.6(0.1–2.6)	5/100(5.0)	0.4(0.1–1.4)

PCI = percutaneous coronary intervention

CI = Confidence Interval

* Odds Ratio for MACE in the Proximal LAD vs. Proximal LCX/RCA group

** Odds Ratio for MACE in the Proximal LAD vs. Non-proximal LAD group

## References

[B1] Valencia J, Bordes P, Berenguer A, Mainar V, Ruiz Nodar JM, Arrarte V (2002). Long-Term Follow-Up of Patients with Proximal Left Anterior Descending Coronary Artery Stenosis Treated with Stent. Rev Esp Cardiol.

[B2] Brambilla N, Repetto A, Bramucci E, Canosi U, Ferrario M, Angoli L, Alello M, Rinaldi M, Kiersy C, Vigano M, Tavazzi L (2005). Directional coronary atherectomy plus stent implantation versus left internal mammary artery bypass grafting for isolated proximal stenosis of the left anterior descending coronary artery. Catheter Cardiovasc Interv.

[B3] Valencia J, Berenguer A, Mainar V, Bordes P, Gomez S, Tello A, Lopez-Aranda MA, Caturla J (2006). Two-Year Follow-up of Sirolimus-Eluting Stents for the Treatment of Proximal Left Anterior Descending Coronary Artery Stenosis. J Interv Cardiol.

[B4] Rodriguez A, Rodriguez Alemparte M, Baldi J, Navia J, Delacasa A, Vogel D, Oliveri R, Fernandez Pereira C, Bernardi V, O'Neill W, Palacios IF (2003). Coronary stenting versus coronary bypass surgery in patients with multiple vessel disease and significant proximal LAD stenosis: results from the ERACI II study. Heart.

[B5] O'Keefe JH, Kreamer TR, Jones PG, Vacek JL, Gorton ME, Muehlebach GF, Rutherford BD, McCallister BD (1999). Isolated left anterior descending coronary artery disease: percutaneous transluminal coronary angioplasty versus stenting versus left internal mammary artery bypass grafting. Circulation.

[B6] Versaci F, Gaspardone A, Tomai F, Proietti I, Ghini AS, Altamura L, Ando G, Crea F, Gioffre PA, Chiariello L (2004). A comparison of coronary artery stenting with angioplasty for isolated stenosis of the proximal left anterior descending coronary artery: five year clinical follow up. Heart.

[B7] Sawhney N, Moses JW, Leon MB, Kuntz RE, Popma JJ, Bachinsky W, Bass T, DeMaio S, Fry E, Holmes DR, Teirstein PS (2004). Treatment of Left Anterior Descending Coronary Artery Disease With Sirolimus-Eluting Stents. Circulation.

[B8] Dangas G, Ellis SG, Shlofmitz R, Katz S, Fish D, Martin S, Mehran R, Russell ME, Stone GW, TAXUS-IV Investigators (2005). Outcomes of paclitaxel-eluting stent implantation in patients with stenosis of the left anterior descending coronary artery. J Am Coll Cardiol.

[B9] Iakovou I, Dangas G, Mehran R, Lansky AJ, Stamou SC, Pfister AJ, Dullum MK, Leon MB, Corso PJ (2002). Minimally invasive direct coronary artery bypass (MIDCAB) versus coronary artery stenting for elective revascularization of the left anterior descending artery. Am J Cardiol.

[B10] Shirai K, Lansky AJ, Mehran R, Dangas GD, Costantini CO, Fahy M, Slack S, Mintz GS, Stone GW, Leon MB (2004). Minimally invasive coronary artery bypass grafting versus stenting for patients with proximal left anterior descending coronary artery disease. Am J Cardiol.

[B11] SOS Investigators (2002). Coronary artery bypass surgery versus percutaneous coronary intervention with stent implantation in patients with multivessel coronary artery disease (the Stent or Surgery trial) a randomised controlled trial. Lancet.

[B12] Ashby DT, Dangas G, Mehran R, Lansky AJ, Narasimaiah R, Iakovou I, Polena S, Satler LF, Pichard AD, Kent KM, Stone GW, Leon MB (2002). Comparison of Clinical outcomes using stents versus no stents after percutaneous coronary intervention for proximal left anterior descending versus proximal right and left circumflex coronary arteries. Am J Cardiol.

[B13] Drenth DJ, Veeger NJ, Grandjean JG, Mariani MA, Van Boven AJ, Boonstra PW (2004). Isolated high-grade lesion of the proximal LAD: a stent or off-pump LIMA?. Eur J Cardiothorac Surg.

[B14] Drenth DJ, Veeger NJ, Boonstra PW (2002). Minimally invasive bypass surgery. N Engl J Med.

[B15] Chobanian AV, Bakris GL, Black HR, Cushman WC, Green LA, Izzo JL, Jones DW, Materson BJ, Oparil S, Wright JT, Roccella EJ, Joint National Committee on Prevention, Detection, Evaluation, and Treatment of High Blood Pressure. National Heart, Lung, and Blood Institute; National High Blood Pressure Education Program Coordinating Committee (2003). Seventh report of the Joint National Committee on Prevention, Detection, Evaluation, and Treatment of High Blood Pressure. Hypertension.

[B16] National Cholesterol Education Program (NCEP) Expert Panel on Detection, Evaluation, and Treatment of High Blood Cholesterol in Adults (Adult Treatment Panel III) (2002). Third Report of the National Cholesterol Education Program (NCEP) Expert Panel on Detection, Evaluation, and Treatment of High Blood Cholesterol in Adults (Adult Treatment Panel III) final report. Circulation.

[B17] Expert Committee on the Diagnosis and Classification of Diabetes Mellitus (2003). Report of the expert committee on the diagnosis and classification of diabetes mellitus. Diabetes Care.

[B18] Goldman L, Hashimoto B, Cook EF, Loscalzo A (1981). Comparative reproducibility and validity of systems for assessing cardiovascular functional class: advantages of a new specific activity scale. Circulation.

[B19] Smith SC, Dove JT, Jacobs AK, Kennedy JW, Kereiakes D, Kern MJ, Kuntz RE, Popma JJ, Schaff HV, Williams DO, Gibbons RJ, Alpert JP, Eagle KA, Faxon DP, Fuster V, Gardner TJ, Gregoratos G, Russell RO, Smith SC, American College of Cardiology/American Heart Association task force on practice guidelines (Committee to revise the 1993 guidelines for percutaneous transluminal coronary angioplasty); Society for Cardiac Angiography and Interventions (2001). ACC/AHA guidelines for percutaneous coronary intervention (revision of the 1993 PTCA guidelines)-executive summary: a report of the American College of Cardiology/American Heart Association task force on practice guidelines (Committee to revise the 1993 guidelines for percutaneous transluminal coronary angioplasty) endorsed by the Society for Cardiac Angiography and Interventions. Circulation.

[B20] Califf RM, Abdelmeguid AE, Kuntz RE, Popma JJ, Davidson CJ, Cohen EA, Kleiman NS, Mahaffey KW, Topol EJ, Pepine CJ, Lipicky RJ, Granger CB, Harrington RA, Tardiff BE, Crenshaw BS, Bauman RP, Zuckerman BD, Chaitman BR, Bittl JA, Ohman EM (1998). Myonecrosis after revascularization Procedures. J Am Coll Cardiol.

[B21] Stone GW, Ellis SG, Cox DA, Hermiller J, O'Shaughnessy C, Mann JT, Turco M, Caputo R, Bergin P, Greenberg J, Popma JJ, Russell ME, TAXUS-IV Investigators (2004). A polymer-based, paclitaxel-eluting stent in patients with coronary artery disease. N Engl J Med.

[B22] Mahmarian JJ, Pratt CM, Boyce TM, Verani MS (1991). The variable extent of jeopardized myocardium in patients with single vessel coronary artery disease: quantification by thallium-201 single photon emission computed tomography. J Am Coll Cardiol.

[B23] Klein LW, Weintraub WS, Agarwal JB, Schneider RM, Seelaus PA, Katz RI, Helfant RH (1986). Prognostic significance of severe narrowing of the proximal portion of the left anterior descending coronary artery. Am J Cardiol.

[B24] Cisowski M, Drzewiecka-Gerber A, Ulczok R, Abu Samra R, Drzewiecki J, Guzy M, Trusz-Gluza M, Bochenek (2004). Primary direct stenting versus endoscopic atraumatic coronary artery bypass surgery in patients with proximal stenosis of the left anterior descending coronary artery – a prospective, randomised study. Kardiol Pol.

[B25] Kurbaan AS, Bowker TJ, Rickards AF (1998). Differential restenosis rate of individual coronary artery sites after multivessel angioplasty: implications for revascularization strategy. CABRI Investigators. Coronary Angioplasty versus Bypass Revascularisation Investigation. Am Heart J.

[B26] Weintraub WS, Kosinski AS, Brown CL, King SB (1993). Can restenosis after coronary angioplasty be predicted from clinical variables?. J Am Coll Cardiol.

[B27] Leborgne L, Cheneau E, Wolfram R, Ajani A, Pakala R, Canos D, Pinnow E, Pichard AD, Satler LF, Waksman R (2002). The proximal location of stenosis in the left anterior descending coronary artery is not a predictive factor of worse outcome in the era of the stent. Cardiovasc Radiat Med.

[B28] Thiele H, Oettel S, Jacobs S, Hambrecht R, Sick P, Gummert JF, Mohr FW, Schuler G, Falk V (2005). Comparison of Bare-Metal Stenting With Minimally Invasive Bypass Surgery for Stenosis of the Left Anterior Descending Coronary Artery: A 5-Year Follow-Up. Circulation.

[B29] Fischman DL, Leon MB, Baim DS, Schatz RA, Savage MP, Penn I, Detre K, Veltri L, Ricci D, Nobuyoshi M, Cleman M, Heuser R, Almond D, Teirstein PSR, Fish D, Colombo A, Brinker J, Moses J, Shaknovich A, Hirshfeld J, Bailey S, Ellis S, Rake R, Goldberg S, for The Stent Restenosis Study Investigators (1994). A randomized comparison of coronary-stent placement and balloon angioplasty in the treatment of coronary artery disease. Stent Restenosis Study Investigators. N Engl J Med.

